# New record of Joffre’s pipistrelle, *Mirostrellusjoffrei* (Chiroptera, Vespertilionidae), in China

**DOI:** 10.3897/BDJ.12.e120923

**Published:** 2024-04-29

**Authors:** Xin Mou, Mei Li, Biao Li, Xiong Luo, Song Li

**Affiliations:** 1 Kunming Natural History Museum of Zoology, Kunming Institute of Zoology, Chinese Academy of Sciences, Kunming, China Kunming Natural History Museum of Zoology, Kunming Institute of Zoology, Chinese Academy of Sciences Kunming China; 2 Conservation Area Management Committee of Guanyin Shan Provincial Nature Reserve, Yuanyang, China Conservation Area Management Committee of Guanyin Shan Provincial Nature Reserve Yuanyang China; 3 Yunnan Key Laboratory of Biodiversity Information, Kunming Institute of Zoology, Chinese Academy of Sciences, Kunming, China Yunnan Key Laboratory of Biodiversity Information, Kunming Institute of Zoology, Chinese Academy of Sciences Kunming China

**Keywords:** first record, *Mirostrellusjoffrei*, morphology, China

## Abstract

**Background:**

*Mirostrellusjoffrei* Thomas, 1915 is the sole species within the genus Mirostrellus and its occurrence is notably rare in China. Despite the recent discovery of four previously unreported specimens from western Yunnan, China (Zaoteng River, 25.31°N, 98.80°E, altitude 1451 m) at the National Museum of Prague in the Czech Republic, comprehensive descriptions or detailed accounts of this species within China have yet to be published.

**New information:**

During a field expedition within the Guanyinshan Provincial Nature Reserve, Yuanyang, Yunnan, China, a bat was captured using a mist net. The specimen was of moderate size, with its ventral surface presenting a golden-brown hue and its dorsal surface ranging from dark brown to obsidian. The forearm measured 37.31 mm, while the fifth digit was conspicuously diminished in comparison to the others. The snout was robust, featuring prominent buccal glands. It was characterised by a well-developed upper frame and a barely visible low sagittal crest. The upper canines possessed well-developed posterior cusps. The dentition comprised two upper and two lower premolars, with the first upper premolar being notably small and the lower molars being distinctly myotodont. These attributes correspond with the known traits of *Mirostrellusjoffrei*. Phylogenetically, the sequence of this specimen clustered with that of *M.joffrei*, forming an independent clade. Through an examination of its morphological characteristics and a phylogenetic analysis of the mitochondrial cytochrome b (Cyt b), cytochrome oxidase subunit I (COI) and recombination activating gene 2 (RAG2) sequences, we identified the specimen as *M.joffrei*, thus confirming the presence of Joffre’s pipistrelle in China.

## Introduction

*Mirostrellusjoffrei* was initially ascribed to the genus *Nyctalus* due to the proportions of its digits (Thomas 1915). Tate (1942) subsequently reclassified the species to the genus *Pipistrellus*, based on the presence of well-developed supraorbital tubercles and minute first premolars (P^2^). Tate (1942) also recognised the *joffrei* group as an offshoot of the *Pipistrellus* genus, suggesting its potential future classification as a subgenus. Ellerman and Morrison-Scott (1951) later re-assigned *joffrei* to the *Nyctalus* genus following its original description. However, following Tate (1942), Hill (1966) retained *joffrei* within the *Pipistrellus* genus, which was later endorsed by Koopman (1973) and Hill and Harrison (1987). Hill and Harrison (1987) concurred with the classification of *joffrei* within the genus *Pipistrellus*. They also categorised *stenopterus* in the subgenus Hypsugo of the same genus, basing this on the distinctive baculum characteristics of the stenopterus compared to other *Pipistrellus* species. Due to the absence of baculum bone data for *anthonyi* and *joffrei*, they also included these species in the subgenus Hypsugo, aligning them with *stenopterus*, based on similarities in their cranial and dental features. Koopman (1994) placed *joffrei*, along with *anthonyi* and *stenopterus*, into *Nyctalus*, while Simmons (2005) subsequently grouped *joffrei* and *anthonyi* under *Hypsugo*. Saikia et al. (2017) merged *anthonyi* with *H.joffrei*, considering them synonymous, based on all available materials at the time. In their detailed analysis of the taxonomic status of *P.stenopterus*, Kruskop et al. (2018) explicitly affirmed that *stenopterus* and *joffrei* belong to markedly distinct phylogenetic lineages within Vespertilioninae, supporting the rationale for distinguishing *joffrei* from the Hypsugo subgenus. Most recently, Görföl et al. (2020) conducted an extensive study of this taxon using phylogenetic, morphological and echolocation data. Their findings revealed a profound divergence between “*H.*” *joffrei* and all other recognied bat taxa, leading to its classification within the novel genus, *Mirostrellus*.

*Mirostrellus* is distributed across the Indomalaya Region, encompassing territories extending from Nepal and the north-eastern regions of India (specifically Sikkim and Meghalaya), through northern Myanmar, to northern Vietnam (Kruskop and Shchinov 2011; Saikia et al. 2017). Four previously unrecorded *Mirostrellus* specimens from western Yunnan, China (Zao Teng He, located at 25.31°N, 98.80°E, altitude 1451 m) were recently discovered by Sergei V. Kruskop at the National Museum of Prague (Czech Republic) (Görföl et al. 2020). Despite this discovery, there remains a notable absence of detailed reports or comprehensive descriptions of this species within China.

During a research expedition in the Guanyinshan Provincial Nature Reserve, Yuanyang, Yunnan, China, a bat specimen was captured. This specimen was distinguished by its distinctive cheek pouches near the ears and nose, possessing a unique fleshy texture and a fifth finger markedly shorter than the third. These morphological features set it apart from typical bats. The distinct nature of this species necessitates additional in-depth research to determine its taxonomic classification.

## Materials and methods

### Sample collection

Fieldwork conducted in May 2023 at the Guanyinshan Provincial Nature Reserve, Yuanyang, Yunnan, China, resulted in the mist-net capture of a bat specimen (field collection number KIZ20230423) in Pinghe, Xiaoxin Street Township (103.00°E, 22.99°N, altitude 2434 m). The specimen was deposited at the Kunming Natural History Museum of Zoology, Kunming Institute of Zoology, Chinese Academy of Sciences (KIZ, CAS).

### Morphological description and measurements

Detailed morphological descriptions were performed on the collected specimen. Ten external measurements were extracted from field records, including: weight (WT), head-body length (HB), tail length (TAIL), ear length (EAR), hind-foot length (HF), forearm length (FA), tibia length (TIBIA), total length of third digit (DIG3), total length of fourth digit (DIG4) and total length of fifth digit (DIG5). Based on the character descriptions in Görföl et al. (2020), cranial and dental measurements were taken with a digital caliper to the nearest 0.01 mm and included: greatest length of skull (GTL): from anterior aspect of first upper incisor to the most prominent point of occipital region; total length of skull (STOTL): from anterior rim of alveolus of first upper incisor to the most projecting point of occipital region; condylobasal length (CBL): from exoccipital condyle to posterior rim of alveolus of first upper incisor; condylocanine length (CCL): from exoccipital condyle to the most anterior part of canine; zygomatic width (ZYW); braincase width (BCW); braincase height (BCH); interorbital width (IOW); mastoid width (MAW); maxillary toothrow length (UCM3L); upper canine-premolar length (UCP4L); upper canine width (UCCW); upper molar greatest width (UM3M3W); upper molar length (UM1M3L); mandible length (MANL); mandibular tooth-row length (LCM3L); lower canine-premolar length (LCP4L); coronoid height (CPH); and lower molar length (LM1M3L).

### Phylogenetic analyses

Genomic DNA was extracted from muscle tissue preserved in anhydrous ethanol using the Ezup Column Animal Genomic DNA Purification Kit (Sangon Biotech, China) in accordance with the manufacturer’s instructions. The complete sequence was amplified and sequenced by utilising primer pairs LGL765: GAAAAACCAYCGTTGTWATTCAACT and LGL766: GTTTAATAAGAATYTYA GCTTTGGG (Bickham et al. 1995) for the cytochrome b (Cyt b) gene; COF: TTCTCAACCAA CCACAAAGACATTGG and COR: TAGACTTCTGGGTGGCCAAAGAATCA (self-designed and optimised) for the cytochrome oxidase subunit I (COI) gene; and 179F: CAGTTTTCTCTAAGGAYTC CTGC and 1458R: TTGCTATCTTCACATGCTCATTGC for the recombination activating gene 2 (RAG2) gene (Stadelmann et al. 2007). Polymerase chain reaction (PCR) was conducted in a 25-μl system comprising 1 μl of template DNA, 1 μl of each primer (10 μM), 1 μl of 10 μM dNTP (mix), 2.5 μl of 10× Taq Buffer (with MgCl_2_), 0.2 μl of 5 U/μl Taq polymerase (Sangon Biotech, China), supplemented with ddH_2_O to a final volume of 25 μl. The PCR protocols entailed an initial denaturation at 95℃ for 5 min, succeeded by 10 cycles of denaturation at 94℃ for 30 s, annealing at 63℃ (with a decrement of 0.5°C per cycle) for 30 s and extension at 72℃ for 30 s, followed by 30 additional cycles of denaturation at 95℃ for 30 s, annealing at 58℃ for 30 s, extension at 72℃ for 30 s, final extension at 72℃ for 10 min and renaturation at 4℃.

The PCR products were visualised by agarose gel electrophoresis and purified using a SanPrep Column DNA Gel Extraction Kit (Sangon Biotech, China). Finally, the purified samples were sequenced using an ABI 3730XL instrument (USA) at Sangon Biotech (Shanghai, China). The obtained sequences were edited and assembled using SeqMan in DNASTAR v.7.1 (DNASTAR Inc., Madison, WI, USA).

A comparative analysis was performed on the Cyt b, COI and RAG2 sequences from our specimen against 12 sequences for each gene from the National Center for Biotechnology Information (NCBI) database. These sequences were identified using GenBank accession numbers provided in Görföl et al. (2020) (Table 1). Alignment was conducted using the ClustalW algorithm (Thompson et al. 1994) with default parameters in MEGA11 (Tamura et al. 2021). Sequences exceeding specific lengths were truncated as follows: COI to 657 bp; Cyt b to 1 140 bp; and RAG2 to 1 151 bp. Genetic distances were calculated using the pairwise distance parameter within the distance module. Uncorrected *P*-distances were obtained by a bootstrap procedure with 1000 replicates. Phylogenetic reconstructions for Cyt b, COI and RAG2 were carried out using Bayesian Inference (BI) with MrBayes v.3.2.6 (Ronquist et al. 2012) executed in PhyloSuite v.1.2.2 (Zhang et al. 2020), employing a partition model with two parallel runs and 2,000,000 generations. The initial 25% of sampled data were discarded as burn-in. ModelFinder (Kalyaanamoorthy et al. 2017) was used to select the best-fit model, based on the Bayesian Information Criterion (BIC): Cyt b, GTR+F+I+G4; COI, HKY+F+I; and RAG2, K2P+G4. Uncorrected *P*-distance and evolutionary analyses were conducted using MEGA11. Data retrieval and organisation were performed using PhyloSuite v.1.2.2 (Zhang et al. 2020) and evolutionary trees were edited with iTOL v.6 (Letunic and Bork 2021).

## Taxon treatments

### Mirostrellus joffrei

Thomas, 1915

D7CCE4E6-97C9-5A46-9963-E6C52E76B3ED

https://www.ncbi.nlm.nih.gov/taxonomy/2741458

#### Materials

- **Type status:** Other material. **Occurrence:** catalogNumber: KIZ20230423; recordedBy: Xin Mou et al.; individualCount: 1; sex: male; lifeStage: adult; occurrenceID: F6735870-3E0E-52CD-BCC3-200C392C38F3; **Taxon:** taxonID: https://www.gbif.org/species/11122810; scientificName: *Mirostrellusjoffrei* Thomas, 1915; kingdom: Animalia; phylum: Chordata; class: Mammalia; order: Chiroptera; family: Vespertilionidae; genus: Mirostrellus; **Location:** country: China; stateProvince: Yunnan; locality: Guanyinshan Nature Reserve, Mt. Guanyin; verbatimElevation: 2434 m; verbatimCoordinates: 22°59.41'N 102°59.91'E; decimalLatitude: 22.99; decimalLongitude: 103.00; georeferenceProtocol: label; **Event:** eventDate: 21-05-23

#### Diagnosis

According to Görföl et al. (2020), "A medium-sized vespertilionid, with a FA of 35.7–40.2 mm. The fifth finger of the wing is shortened (on average 20 mm shorter than the fourth finger) and the pelage is sparse and velvety. The supraorbital tubercles are well-developed, protruding for 1.47–1.76 mm measured from the lachrymal opening; the sagittal crest is barely visible, being only approximately 0.1 mm high. The upper canine is characterised by a developed posterior secondary cusp. The taxon has two upper and lower premolars and its lower molars are myotodont".

## Analysis

### Morphological characteristics

**Body**

**Figure 1.**
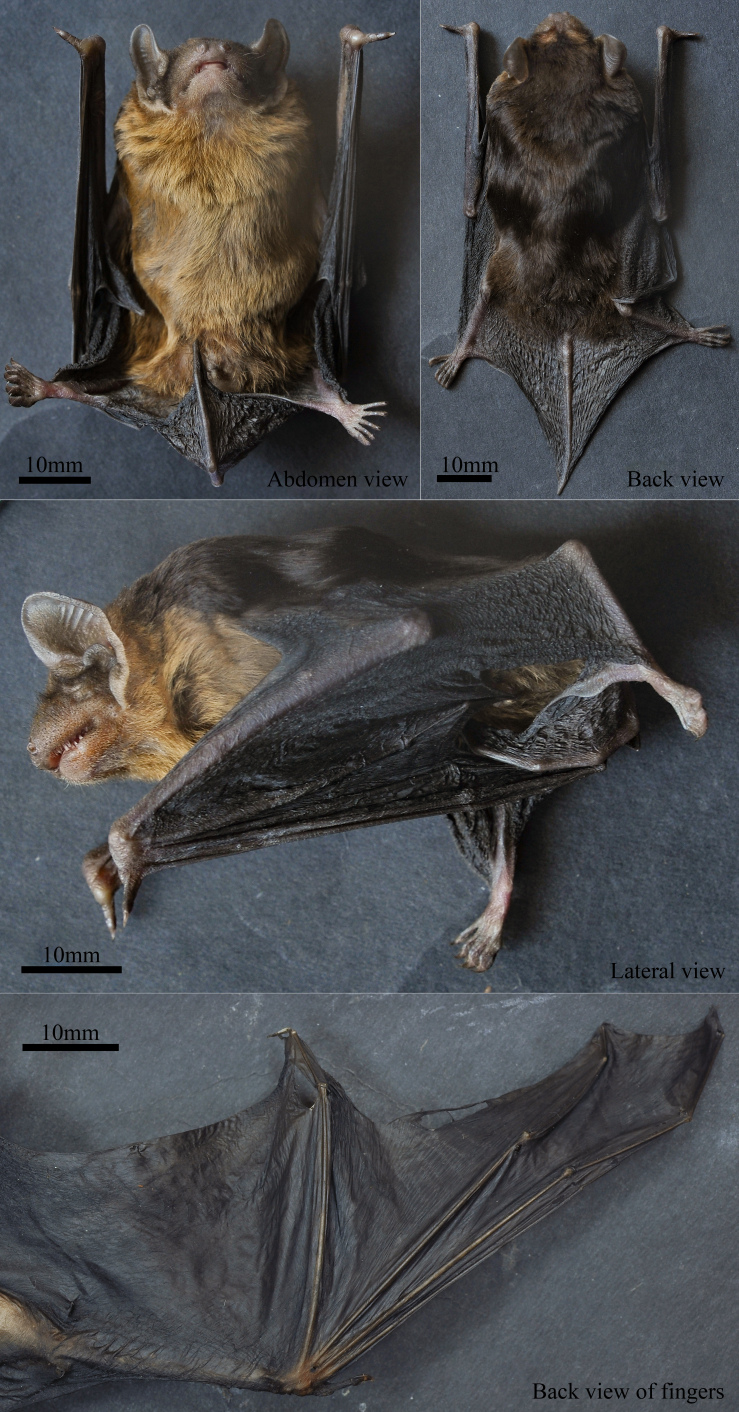
Photo of *Mirostrellusjoffrei* specimen (collection number KIZ20230423) from this study.

Moderate size, with head-body length of 54.73 mm, tail length of 42.56 mm and forearm length of 37.31 mm (Table 2). Mouth and nose round, thick and swollen in appearance, forming prominent cheek pouches. Several long whiskers present on sides of nose. Ears fleshy, large and circular in shape; auricle short, wide, extending below snout; earlobe incompletely developed, exceptionally small, extending upwards; inner margin recessed, outer margin pronounced and elevated (Fig. 1). Wings long and narrow, displaying common characteristics of bats capable of fast flight (Fig. 1). Third finger (64.10 mm) 22.89 mm longer than fifth finger (41.21 mm), consistent with description by Görföl et al. (2020) (Table 2). Tail and wing membranes with uniform black hue; tip of tail bone protruding approximately 2 mm beyond tail membrane; wing membrane attached to ankle bone (Fig. 1). Specimen male, external morphology evident, penis bone absent upon dissection.

**Fur**

Fur soft and lustrous in appearance, moderate length. Overall colour of dorsal fur blackish-brown and glossy, ventral fur golden-brown with clear demarcation when viewed from side. Hair at tip of snout short and sparse. Middle of lower jaw greyish-white, sides golden-brown, upper jaw blackish-brown with brown spots. Hair almost non-existent on forearms, tibia and feet (Fig. 1).

**Skull**

**Figure 2.**
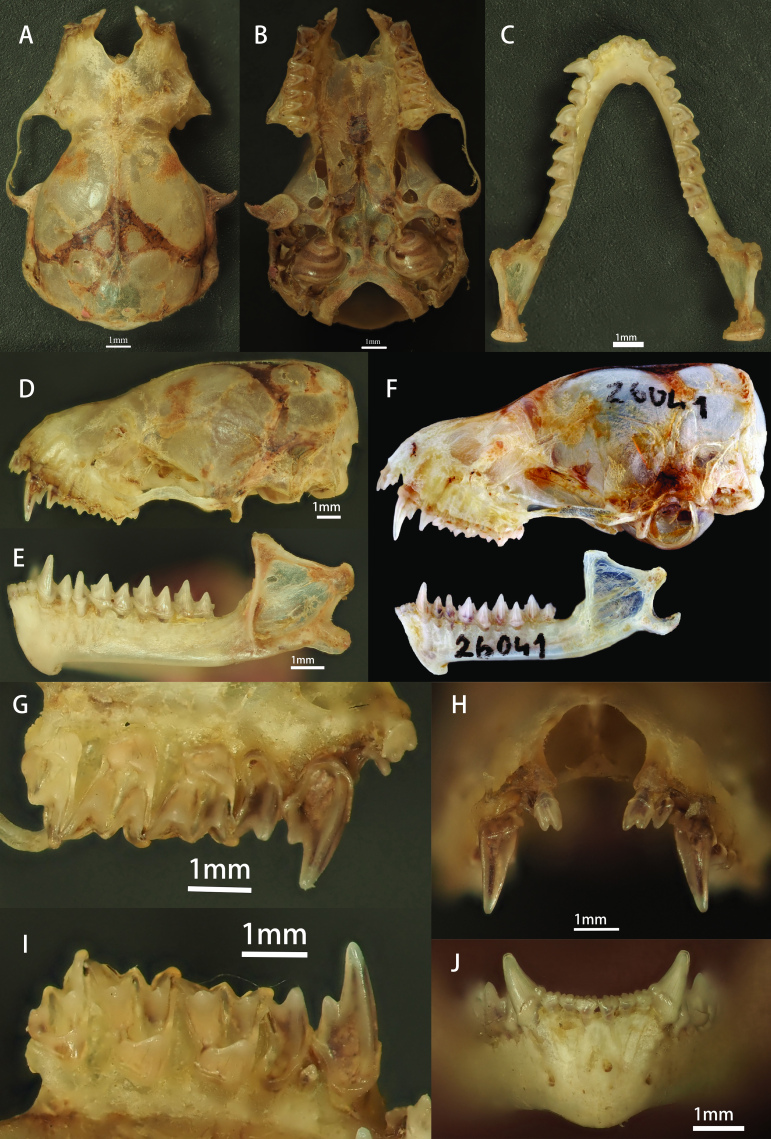
Cranial morphology of *Mirostrellusjoffrei* (KIZ20230423 and HNHM 26041 from Mu Cang Chai, Vietnam). **A** Dorsal view of skull; **B** Ventral view of skull; **C** Dorsal view of mandible; **D** Left-side view of cranium; **E** Lateral view of the left mandible; **F** Lateral view of skull of *Mirostrellusjoffrei* from Mu Cang Chai, Vietnam (HNHM 26041) (Görföl et al. 2020); **G** Medial view of left maxillary dental arch; **H** Maxillary incisors and canines; **I** Medial view of right maxillary dental arch; **J** Mandibular incisors and canines.

Skull moderate in size, compact in appearance, with short and wide snout (Fig. 2A and B). When viewed from the side, there is a gradual upward slope from front of nose to back of forehead, with slight indentation in centre of forehead and well-developed supraorbital tubercles, protruding noticeably beyond outline of skull. Sagittal crest low and slightly protruding from skull, but not prominent (Fig. 2D). Entire cranial cavity rectangular in shape, infraorbital foramen prominent. Middle part of zygomatic arch slightly protruding upwards (Fig. 2D). When viewed from above the aspect of the cranium, the cranial cavity appears slightly rounded, with slight inward concavity in the middle of the zygomatic arch (Fig. 2A and B). Mandible slightly robust, with relatively high coronoid process. Area between coronoid process and condyle relatively smooth, lacking distinct concavity. Line connecting condyle and angle of mandible perpendicular to mandibular body. Angle of mandible straight, without curvature, forming approximate 30-degree angle with elongation line of mandibular body (Fig. 2E).

**Dentition**

Dental formula I 2/3, C 1/1, P 2/2 and M 3/3 (Fig. 2B, C, H and I). Upper incisors wide and short, exhibiting distinct bicuspid shape, noticeably taller than outer incisors (Fig. 2H). Upper canines larger, with distinct cusp located in middle of posterior edge (Fig. 2D). First upper premolar (P^2^) tiny, located between upper canine and second upper premolar (P^3^) in right dental arcade (Fig. 2I), not visible to the naked eye in left dental arcade (Fig. 2G). P^3^ well-developed, approximately half the size of the canine (Fig. 2D). Lower incisors aligned in a row (Fig. 2J), lower canines slender, lacking cusp (Fig. 2E). Height of first lower premolar (P_2_) slightly lower than that of second lower premolar (P_4_) (Fig. 2E), lower molars clearly myotodont (Fig. 2B). These characteristics are consistent with descriptions by Thomas (1915), Saikia et al. (2017) and Görföl et al. (2020) for this species.

### Phylogenetic analysis

**Figure 3.**
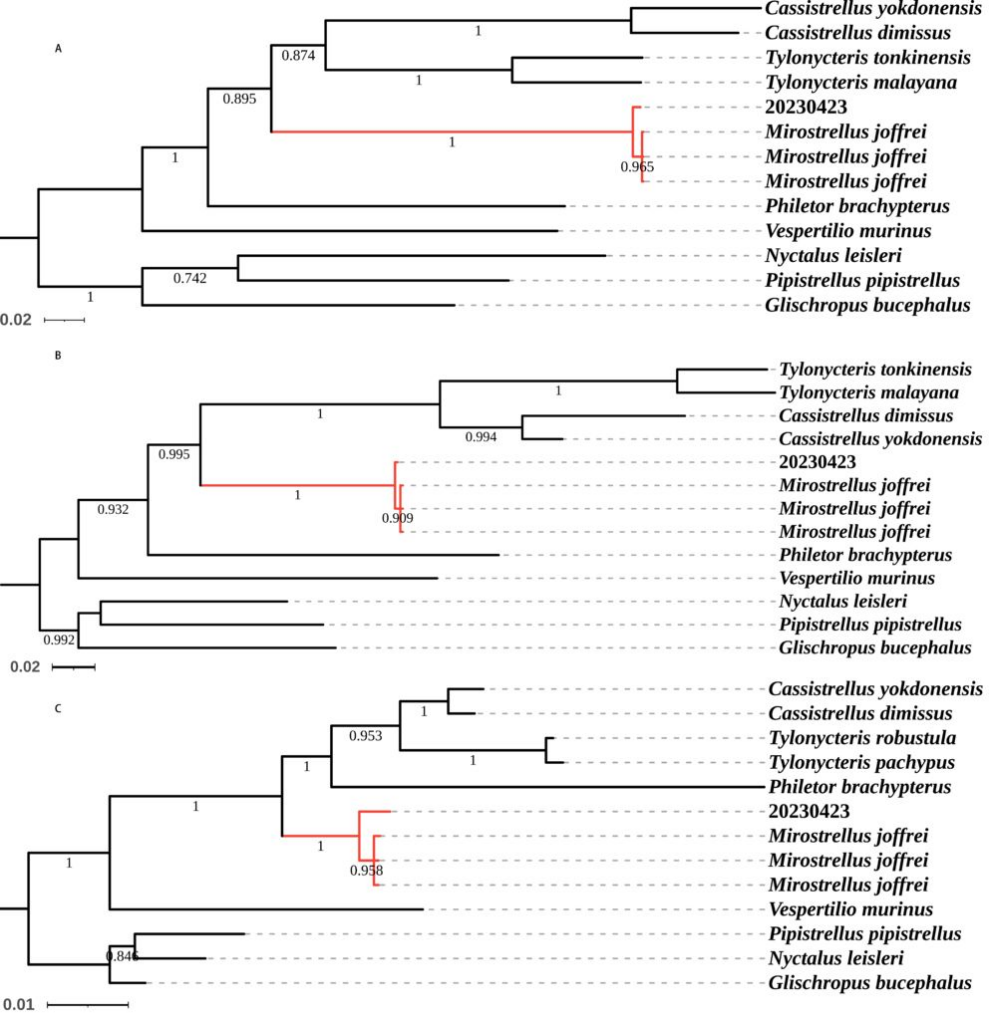
Phylogenetic tree depicting relationships amongst selected vespertilionid bat species, constructed using Bayesian Inference. *Glischropusbucephalus*, *Pipistrelluspipistrellus* and *Nyctalusleisleri* were designated as the outgroup. Posterior probabilities are shown adjacent to nodes (nodes with posterior probabilities < 0.50 are not labelled). Analysis based on sequences from: **A** Cyt b gene; **B** COI gene; **C** RAG2 gene.

The NCBI database alignment for the Cyt b, COI and RAG2 sequences classified the specimen as *M.joffrei*, with sequence identities of 99.47%, 99.85% and 99.57%, respectively. The uncorrected *P*-distances between our specimen and *M.joffrei* for the Cyt b, COI and RAG2 genes were notably small, significantly lower than those observed for other species, as outlined in Tables 3, 4. In phylogenetic analysis, our specimen clustered with *M.joffrei*, forming a monophyletic group supported by a posterior probability of 1 (Fig. 3A, B and C).

## Discussion

The specimen demonstrated a conspicuous colouration contrast between its dorsal and ventral fur; the ventral fur exhibited a lighter, golden-brown hue, whereas the dorsal fur appeared darker, with a lustrous brown-black tint. The snout was short and broad, with apparent swelling at the snout and nasal region, forming distinct cheek pouches. Its wings were narrow and long, with a free tail. The fifth finger was markedly reduced, especially compared to the third finger. Cranially, the specimen featured well-developed supraorbital ridges and a low, barely visible sagittal crest. The upper canines exhibited a prominent secondary cusp, extending halfway up the main cusp. The lower molars were clearly myotodont, characterised by a connection between the hypoconulid and hypoconid via the postcristid. Two upper premolars were observed, with the notably minute P^2^ being positioned between the canine and P^3^, orientated towards the lingual side. Given its small size and lingual inclination, P^2^ was only visible from the lingual aspect and not from the labial aspect. From the lingual perspective, P^2^ was visibly on the right, but was not visually identifiable on the left. These characteristics align with the descriptions of *M.joffrei* provided by Thomas (1915), Saikia et al. (2017) and Görföl et al. (2020). Molecular analyses, including genetic distance and phylogenetic tree analyses, unequivocally placed the specimen within *M.joffrei*.

Although our specimen adhered closely to the diagnostic characteristics of *M.joffrei*, subtle differences were evident that warrant closer examination. The measurement data of this study are relatively smaller compared to the literature records. This could be due to the following reasons: 1, Errors in measurement by different individuals; 2, There is no description of sexual dimorphism in *M.joffrei* in literature, but sexual dimorphism does exist in Vespertilionidae, typically characterised by smaller size in males and larger size in females. The smaller size of our specimens may be due to sexual dimorphism; 3, Our specimens were collected at an altitude of 2434 m, which is higher than the altitude range recorded in literature. This difference may be due to varying geographical and climatic environments. We only collected one specimen and, if there are more sample materials in the future, we can conduct a more in-depth study. Specifically, when viewed from the lateral aspect, our specimen exhibited a more pronounced curvature of the zygomatic arch compared to specimen HNHM 26041 from Mu Cang Chai, Vietnam. Furthermore, our specimen featured a slight curvature in the retroarticular process, while the angle remained straight. In contrast, HNHM 26041 exhibited a straight retroarticular process with a posteriorly concave angle (Fig. 2D and F). Additionally, the image of specimen ZMMU S-186691, as illustrated in the work of Görföl et al. (2020), presents a similar cranial morphology to the photograph of HNHM 26041. Notably, the cranial photograph utilised for comparison in our article (HNHM 26041) and the illustration derived from the specimen (ZMMU S-186691) in Görföl et al. (2020) do not correspond to the sources of the molecular comparison data (IEBR VN16-170, HNHM 26037 and HNHM 26040). We are currently uncertain if the skulls of IEBR VN16-170, HNHM 26037 and HNHM 26040 exhibit differences similar to those noted in our specimen. Should there be variations in these three specimens relative to ours, it will be necessary to assess whether these are due to individual differences or if they suggest a subspecies classification. Conversely, the absence of differences would warrant further investigation into the relationship amongst these specimens. As there are no cranial photographs available for these three specimens and the aforementioned differences do not pertain to the diagnostic features of this species, we have opted to publish this specimen as a new record of *M.joffrei* in China.

As the sole recognised species within the genus *Mirostrellus*, *M.joffrei* demonstrates a distribution range spanning from Nepal and the north-eastern regions of India (Sikkim, Meghalaya) to the northern reaches of Myanmar, further extending to northern Vietnam (Saikia et al. 2017). Four previously unreported *Mirostrellus* specimens from western Yunnan, China (Zaoteng River, 25.31°N, 98.80°E, altitude 1451 m), were recently discovered at the National Museum of Prague (Czech Republic) (Görföl et al. 2020) by researchers affiliated with the Zoological Museum at Moscow State University. Despite this discovery, there are no detailed reports or comprehensive descriptions of this species within China. In the current study, we identified a bat specimen captured in Yunnan, China as *M.joffrei*, based on morphological characteristics and phylogenetic relationships, thereby confirming the presence of the species in China and formally documenting it as a new record for China.

Our specimen was collected in Guanyinshan Nature Reserve, Yuanyang County, Yunnan Province, China, situated in the southern extension of the Ailao Mountain Range. It was obtained from original evergreen broadleaf forest at an altitude of 2434 m. The canopy of the forest is dense, with abundant shrubs and small streams passing through. No caves or rock crevices were noted in the area; however, tree hollows were evident in some ancient trees (diameters of approximately 1 m), suggesting a greater likelihood that these bats dwell in such hollows or under the dense canopy. According to Görföl et al. (2020), the collection records of this species predominantly originate from higher altitudes, ranging from 575 to 2038 m, primarily above 1000 m. Our specimen was collected at an altitude of 2434 m, surpassing the previously recorded highest altitude by nearly 400 m. In terms of distribution, *M.joffrei* has been reported from the Kachin Mountains in Myanmar to the Hoàng Liên Son Range in Vietnam. The discovery of the specimen in the Guanyinshan Nature Reserve in Yuanyang, which falls within the southern Ailao Mountains, suggests the potential presence of this species in the Kachin Mountains, Hoàng Liên Son Range and Ailao Mountains within China. Given its elusive nature and its remarkable agility and swift flying abilities, detecting *M.joffrei* poses a considerable challenge and our knowledge, regarding its population, habitat preferences and ecology, remains limited. Based on observations of the species' habitat during the survey, it is recommended to protect it by maintaining the original state of its habitat, prohibiting grazing, cultivation and logging, which seriously impact its habitat within its active area and reducing other human activities. In conclusion, the most prudent approach to conserving this rare bat species involves the preservation of its habitat.

## Supplementary Material

XML Treatment for Mirostrellus joffrei

## Figures and Tables

**Table 1.** Species (with GenBank accession numbers) used for phylogenetic analysis and calculation of uncorrected *P*-distances.

Species	Cyt b	COI	RAG2	Reference
*Cassistrellusdimissus*	MG194436	MG194430	GU328057	Lack et al. (2010); Ruedi et al. (2018)
*Cassistrellusyokdonensis*	MG194435	HM540266	MG194433	Francis et al. (2010); Ruedi et al. (2018)
*Glischropusbucephalus*	KR612334	KR612331	MH753142	Csorba et al. (2015); Görföl and Csorba (2018)
*Mirostrellusjoffrei* 1	MN813973	MN813969	MN813977	Görföl et al. (2020)
*Mirostrellusjoffrei* 2	MN813974	MN813970	MN813978	Görföl et al. (2020)
*Mirostrellusjoffrei* 3	MN813975	MN813971	MN813979	Görföl et al. (2020)
KIZ20230423	PP719688	PP718733	PP719689	This study
*Nyctalusleisleri*	JX570901	JF443043	HM561657	Kruskop et al. (2012); Heaney et al. (2012); Roehrs et al. (2010)
*Philetorbrachypterus*	KX429688	HM541204	JX570922	Francis et al. (2010); Lim et al. (2016); Heaney et al. (2012)
*Pipistrelluspipistrellus*	AJ504443	JF443078	HM561662	Kruskop et al. (2012); Stadelmann et al. (2004); Roehrs et al. (2010)
*Tylonycterismalayana*	KX496401	KX496402		Tu et al. (2018)
*Tylonycterispachypus*			JX570928	Heaney et al. (2012)
*Tylonycterisrobustula*			HM561673	Roehrs et al. (2010)
*Tylonycteristonkinensis*	KX496441	KX496442		Tu et al. (2018)
*Vespertiliomurinus*	MN813976	MN813972	MN813980	Görföl et al. (2020)

**Table 2.** Weight (in g) and external and cranial measurements (in mm) of *Mirostrellusjoffrei*.

Character	KIZ20230423 (this study)	Specimens of Görföl et al. (2020)		Shillong specimen ZSI V/M/ERS/292. Saikia et al. (2017)	The original description by Thomas (1915)
		mean ± SD	min – max (n)		
WT	12.7	16.0 ± 1.95	13.0 – 19.0 (18)		
HB	54.73			61	56
TAIL	42.56			37	39
EAR	14.00			13.2	
HF	8.8			8.3	8
FA	37.31	38.6 ± 1.08	35.7 – 40.2 (28)	40.2	39
TIBIA	15.53	15.4 ± 0.74	13.8 – 16.4 (12)	15.6	15
DIG3	64.10			66	
DIG4	52.82			57.5	
DIG5	41.21			46	
GTL	15.37	15.33 ± 0.27	14.86 – 15.77 (12)		15
STOTL	15.02	14.91 ± 0.30	14.47 – 15.35 (15)	15.1	14.2
CBL	13.69			14.8	
CCL	14.00	14.21 ± 0.24	13.65 – 14.72 (17)	13.98	
UCM3L	5.15	5.19 ± 0.06	5.12 – 5.30 (16)	5.18	5.1
UM3M3W	7.37	7.08 ± 0.15	6.79 – 7.34 (18)	7.23	
UCCW	5.3	5.15 ± 0.11	4.96 – 5.34 (16)	5.01	
UM1M3L	3.31			3.6	
ZYW	10.86	10.47 ± 0.31	9.95 – 10.99 (13)	10.09	10.5
IOW	4.65	4.68 ± 0.17	4.34 – 5.02 (20)	4.7	4.5
MAW	8.72	8.90 ± 0.18	8.58 – 9.28 (17)	9.2	
BCW	8.23	8.04 ± 0.25	7.64 – 8.58 (17)	7.96	8.2
BCH	5.97	5.57 ± 0.19	5.36 – 6.00 (10)	5.9	
UCP4L	1.98	2.05 ± 0.07	1.90 – 2.18 (13)	2	
MANL	10.52	10.89 ± 0.25	10.53 – 11.45 (19)	10.9	
LCM3L	5.22	5.55 ± 0.10	5.29 – 5.70 (19)	5.29	5.5
LCP4L	1.76	1.66 ± 0.17	1.44 – 1.80 (4)	1.78	
CPH	3.82	3.66 ± 0.10	3.54 – 3.90 (14)	3.8	
LM1M3L	3.74			3.8	

**Table 3.** Uncorrected *P*-distances (%) amongst sequences. Lower left values correspond to Cyt b dataset, upper right values correspond to COI dataset.

	Species	1	2	3	4	5	6	7	8
1	20230423		0.15	0.15	0.15	15.83	15.83	16.80	18.85
2	*Mirostrellusjoffrei* 1	0.70		0.00	0.00	15.68	15.68	16.96	19.01
3	*Mirostrellusjoffrei* 2	0.70	0.00		0.00	15.68	15.68	16.96	19.01
4	*Mirostrellusjoffrei* 3	0.70	0.00	0.00		15.68	15.68	16.96	19.01
5	*Tylonycterismalayana*	17.38	17.37	17.37	17.37		7.15	17.12	16.93
6	*Tylonycteristonkinensis*	17.12	17.11	17.11	17.11	9.21		17.92	17.57
7	*Cassistrellusyokdonensis*	18.26	18.16	18.16	18.16	16.67	17.81		10.08
8	*Cassistrellusdimissus*	18.82	19.06	19.06	19.06	17.01	17.97	8.93	

**Table 4.** Uncorrected *P*-distances (%) amongst RAG2 sequences.

	Species	1	2	3	4	5	6	7	8
1	20230423								
2	*Mirostrellusjoffrei* 1	0.52							
3	*Mirostrellusjoffrei* 2	0.26	0.00						
4	*Mirostrellusjoffrei* 3	0.26	0.00	0.00					
5	*Tylonycterispachypus*	5.63	5.49	5.37	5.37				
6	*Tylonycterisrobustula*	17.46	17.29	17.21	17.21	0.14			
7	*Cassistrellusyokdonensis*	4.00	3.82	3.67	3.67	2.95	15.03		
8	*Cassistrellusdimissus*	4.00	3.82	3.67	3.67	2.53	14.77	0.70	
